# Towards a low carbon ASEAN: an environmentally extended MRIO optimization model

**DOI:** 10.1186/s13021-022-00213-x

**Published:** 2022-09-07

**Authors:** Adrianus Amheka, Hoa Thi Nguyen, Krista Danielle Yu, Robert Mesakh Noach, Viknesh Andiappan, Vincent Joseph Dacanay, Kathleen Aviso

**Affiliations:** 1 Department of Mechanical Engineering, State Polytechnic of Kupang, Kupang City, Indonesia; 2 Division of Sustainable Energy and Environmental Engineering, Osaka University, Osaka, Japan; 3 School of Economics, De La Salle University, Manila, Philippines; 4 Department of Business Administration, State Polytechnic of Kupang, Kupang City, Indonesia; 5 Faculty of Engineering, Computing and Science, Swinburne University of Technology, Kuching, Sarawak Malaysia; 6 Department of Chemical Engineering, De La Salle University, Manila, Philippines; 7 School of Engineering and Physical Sciences, Heriot-Watt University Malaysia, Putrajaya, Malaysia

**Keywords:** Low carbon economy, Optimization, Environmentally extended MRIO

## Abstract

**Background:**

Economic growth is dependent on economic activity, which often translates to higher levels of carbon emissions. With the emergence of technologies that promote sustainable production, governments are working towards achieving their target economic growth while minimizing environmental emissions to meet their commitments to the international community. The IPCC reports that economic activities associated with electricity and heat production contributed most to GHG emissions and it led to the steady increase in global average temperatures. Currently, more than 90% of the total GHG emissions of the ASEAN region is attributable to Indonesia, Malaysia, the Philippines, Thailand, and Vietnam. These regions are expected to be greatly affected with climate change. This work analyzes how ASEAN nations can achieve carbon reduction targets while aspiring for economic growth rates in consideration of interdependencies between nations. We thus develop a multi-regional input–output model which can either minimize collective or individual carbon emissions. A high-level eight-sector economy is used for analyzing different economic strategies.

**Results:**

This model shows that minimizing collective carbon emissions can still yield economic growth. Countries can focus on developing sectors that have potentials for growth and lower carbon intensity as new technologies become available. In the case study examined, results indicate that the services sector, agriculture, and food manufacturing sector have higher potential for economic growth under carbon reduction emission constraints. In addition, the simultaneous implementation of multiple carbon emission reduction strategies provides the largest reduction in regional carbon emissions.

**Conclusions:**

This model provides a more holistic view of how the generation of carbon emissions are influenced by the interdependence of nations. The emissions reduction achieved by each country varied depending on the state of technology and the level of economic development in the different regions. Though the presented case focused on the ASEAN region, the model framework can be used for the analysis of other multi-regional systems at various levels of resolution if data is available. Insights obtained from the model results can be used to help nations identify more appropriate and achievable carbon reduction targets and to develop coordinated and more customized policies to target priority sectors in a country. This model is currently limited by the assumption of fixed technical coefficients in the exchange and interdependence of different regions. Future work can investigate modelling flexible multi-regional trade where regions have the option of substituting goods and products in its import or export structure. Other strategies for reducing carbon emission intensity can also be explored, such as modelling transport mode choices, or establishing sectors for waste management. Hybrid models which integrate the multi-regional input–output linear program model with data envelopment analysis can also be developed.

## Background

An increasing trend in greenhouse gas (GHG) emissions has been observed since the 1900s and it has been linked primarily to fossil fuel combustion and industrial activities. In 2014, the Intergovernmental Panel on Climate Change (IPCC) reported that economic activities associated with electricity and heat production contributed most to these emissions (e.g. 25% of the global emissions). China was identified as the highest emitting country accounting for 30% of the global emissions (IPCC 2014). The increase in atmospheric GHG concentration has led to the steady increase in global average temperatures. Projections indicate that a global average temperature rise of 2 °C from the pre-industrial period is likely to occur by 2100 if no concrete actions to reduce GHG emissions are implemented [1]. This scenario can lead to serious consequences such as sea level rise [2], water shortages, increased rainfall, and crop failure [3] which will have a significant impact on livelihoods and economic performance.

The Association of Southeast Asian Nations or ASEAN was established in Bangkok in 1967. The ASEAN includes 10 countries namely Brunei, Cambodia, Indonesia, Laos, Malaysia, Myanmar, the Philippines, Singapore, Thailand, and Vietnam. Though the region currently does not contribute significantly to GHG emissions, its role in climate change mitigation should be monitored and examined because of the region’s recent rapid industrialization and economic growth [4]. Approximately 90% of the total GHG emissions of the ASEAN region is attributable to five countries namely, Indonesia, Malaysia, the Philippines, Thailand, and Vietnam [5]. In addition, the region is expected to be greatly affected with climate change with majority of its economic activities heavily reliant on agriculture and coastal activities.

Globally, countries have identified their Nationally Determined Contributions (NDC) towards reducing GHG emissions and have reaffirmed their commitment to the Paris Agreement in 2018 in the recent conference of parties (COP26) held last November 2021. In this forum, ASEAN member states reported the achievement of a 21% energy intensity reduction in the region [6]. However, achieving these targets based on identified strategies remains a question. [7] examined the commitment of six ASEAN countries with particular focus on the strategies for the energy and transport sector. They found that though the countries have set forth several policies towards meeting the goal, few of them have been quantified or evaluated for their potential to succeed. Another challenge for the region is in maintaining the growth of the economy amidst emission reduction efforts. [8] analyzed the driving forces behind the CO_2_ emission using the log mean Divisia index (LMDI) and found that carbon density effect, per capita GDP effect, and the population effect are the main contributors towards increasing emissions, while energy intensity effect contributed towards reducing emissions in the ASEAN region [9] on the other hand, examined the dynamic relationship between energy, CO_2_ emissions, and economic growth of the ASEAN region. [10] showed that an alternative approach through consumption-based accounting can yield different results compared to the traditional production-based approach.

Numerous approaches towards emission reduction have been adapted. Indonesia and the Philippines have implemented climate change budget tagging, wherein each branch of government, down to the local level indicates whether their expenditure contributes towards sustainability [11, 12]. Indonesia’s NDC has set an unconditional GHG emission reduction target of 29% and conditional reduction target of up to 41% from the business as usual (BAU) scenario by 2030 through finance, technology transfer, technology development, and capacity building which will cover among others the sectors of energy, agriculture, industry, waste, and forestry [13–15]. The Philippines’ NDC is committed to reduce its GHG emissions by 75% in 2030 relative to its BAU scenario [16, 17] for the five leading sectors (i.e. energy, transport, waste, forestry and industry) [18]. The Philippine government mitigation strategy focuses on research and capacity development, while adaptation focuses on being more climate and disaster-resilient. External assistance is still necessary to support the development and adoption of most technologies to improve adaptive capacities and resilience. Both Indonesia and the Philippines are putting more focus on forestry, agriculture, and clean power generation. Indonesia also has plans to phase out coal by 2040, if sufficient technical and financial support from external parties is available [19]. For Vietnam, its NDC commitment is to reduce its GHG emissions by 8% by 2030 with a potential increase to 25% compared to BAU if the initiative is supported through bilateral and multilateral cooperation [20]. The efforts are expected to reduce the emission intensity per unit of GDP by 20–30% compared to 2010 levels. The intention is to increase the proportion of new and renewable energy (RE) in energy production and consumption. This strategy will cover and affect the sectors of industry and transportation, agriculture, land use, land-use change and forestry (LULUCF), and waste [21, 22]. Vietnam also committed to reduce CO_2_ at COP26 and emphasized that renewable energy will play an important role in this effort. Thailand's emissions represent around 0.84% of global emissions. Thailand’s NDC is targeted to reduce its GHG emissions by 20% from the BAU level by 2030 [23]. It has the potential to be reduced beyond the target through technology development and financial resources, while its adaptation action focuses on water resources management, agriculture, forest, and tourism [23]. For Cambodia, the INDC is set at 16% by 2030 which can be achieved through harnessing the renewable energy potential in power generation and promoting energy efficiency for end users. It has been identified that Cambodia is rich in renewable energy resources however, it lacks policies that will promote the development of such resources. Thus, the government has been exploring incentives such as feed-in tariffication, renewable portfolio standards, and net metering to promote expansion in the sector [24]. Malaysia and Singapore have NDC targets imposed on the sectors namely energy, industry, agriculture, land use, and waste. For Malaysia, its GHG emission contributed around 0.6% to the global emissions, and its NDC unconditional reduction target for 2030 is at 35%, which can be increased to 45% if given international support [25]. Malaysia will focus on climate finance, and technology development [26, 27]. Singapore, a developed country accounts for approximately 0.11% of global emissions and 2.2% of global trade. Singapore’s NDC target is to reduce GHG emissions intensity by 36% by 2030 [28]. Singapore has designed a multi-sectoral plan to mitigate the carbon emissions not only in power generation but also tightening standards for household appliances, promoting green buildings and more efficient transportation [29].

Based on the description of the NDC targets of ASEAN countries, there are similarities on the economic sectors that are expected to contribute towards GHG emission reductions (i.e., energy, industry, waste, agriculture, and forestry), showing the similarities in characteristics among ASEAN developing countries. The identified sectors have a major influence on the survival of the community as a basis for livelihood [30]. With global energy policy trends directed towards addressing SDG 7 on affordable and clean energy, initiatives have focused on identifying strategies to improve energy efficiency, supporting low-carbon technologies, and increasing RE penetration. Similar strategies are being explored in the ASEAN region and will be the focus of discussion in the case studies presented in this work. In 2019, the ASEAN reached a 24.4% reduction in energy intensity compared to 2005 levels, exceeding the 20% reduction target set for 2020. In 2017, the contribution of RE to the energy mix of total primary energy supply within the ASEAN reached 14.3% of the targeted 23% share in TPES by 2025 [31]. There are several strategies that can be implemented to achieve low-carbon targets, and these can be applied at various levels of the economy. Work on how to decarbonize specific industries such as iron and steel manufacturing have been reviewed recently by [32], deployment of smart energy systems at a regional or national level has also been proposed [33, 34], while [35] focused on analyzing national policies and how they relate to the emissions resulting from international supply chains. On a national level, the deployment of measures to lower GHG emissions may result in constraining the growth in economy of some countries especially if the most productive sectors are the most pollutive ones. Thus, it is necessary for governments and relevant stakeholders to evaluate how carbon emission reduction strategies influence economic growth [36].

Early work on assessing the technological capability of ASEAN countries [37] where ASEAN performance was benchmarked against more productive nations. The use of Data Envelopment Analysis (DEA) [38] which was developed for defining various forms of efficiency through the selection of appropriate input and output performance indicators, has also been used for examining the environmental performance of economies. Examples include relating environmental sustainability and economic growth [39] (and assessing carbon emissions efficiency for sectors of the economy [33]. Such approaches are useful for identifying empirically efficient examples (i.e., decision-making units (DMUs)) in the dataset and determining relevant factors which favor or deter efficiency. However, the DEA is unable to represent interactions between DMUs which can potentially influence over-all performance of an economy. Such interactions can modelled using input–output (IO) analysis [40]. The national IO model provides a tool for capturing the interconnectedness among different economic sectors [41]. The national IO analysis can be extended to quantify other aspects within the economic system. For example, [42] made use of inoperability input–output model introduced by [43] coupled with the vulnerability indictors proposed by [44] to develop a multi-criteria framework for evaluating disaster vulnerability due to deployment of a biofuel regulation while [45] examine prioritizing economic sectors for post-pandemic recovery in a country scale. The extended IO model that considers environmental burdens, has been applied for optimizing supply chains in consideration of water footprint constraints [46] in optimizing multi-regional bioethanol supply chains with fuzzy multi-objectives [47]; and in identifying the role of economic sectors as pollution producers [48]. Besides these, the national IO analysis was also extended to link with structural decomposition analysis to uncover the main socio-economic driving factors on the increase of CO_2_ emission within the Vietnamese economy [49] and on the decrease of toxic chemical releases in the Japanese economy [50]. The national IO analysis has also been extended to MRIO analysis to allow for the examination of global or international supply chains. This model is useful to evaluate economic-environmental impacts embodied in international trade among nations because of the growth of globalized markets. For example, [51] proposed a method using MRIO to estimate CO_2_ emissions embodied in the trade among 87 countries. [52] provided a review on studies of MRIO on the basics of consumption-based emissions and resource accounting, while [53] estimated undated carbon footprint using global trade analysis project database (GTAP-MRIO). [54] used MRIO to quantify carbon and water footprint for both production-based and consumption-based emissions while [55] made use of an environmentally-extended model to track carbon emissions in Denmark also using production and consumption-based perspectives.

The capability of the IO model is further strengthened if it is coupled with systematic optimization tools such as linear programing (LP) [56]. A comprehensive review of IO-LP indicates the advantages of this hybrid approach in comparison to the conventional IO model [36]. The IO-LP can identify the proper productivity of economic activities to find the optimal solutions for a given objective function (i.e., maximize gross domestic product) while maintaining the balance of sectoral productivity levels. Furthermore, IO-LP may provide a more comprehensive evaluation of effective production possibilities and economic impact from implementing potential regulations and allow for the study of trade-offs among conflicting objectives [36]. For example, IO-LP has been used for quantifying the macroeconomic costs due to an implementation of CO_2_ reduction policy [57]; and for evaluating the trade-offs among economic, environmental, and energy objectives for Brazil’s economy [58]. This approach was used to maximize the gross domestic output in the Greek economic system under constraints of GHG, energy and final demands [59]; and then extended to couple with the impact of solid waste [60]. [16] developed an IO-LP based on fractional programing, which aims to minimize the carbon intensity in the Philippine economy in consideration of economic development and climate target as given in the Philippine’s NDC. Similar works was also carried out by [22] to minimize the total GHG emissions of the Vietnamese economy. Meanwhile, [22] used IO-LP for mapping low-carbon scenarios and quantifying the reduction on human health damage by applying different technological improvement methods. However, the application of LP for MRIO models is still limited, a keyword search in the Scopus database using the keywords [TITLE-ABS-KEY (multi AND regional AND input–output)] AND [(optimization)] AND (linear AND program) only yielded 16 documents. Part of the difficulty stems from data requirement and model complication. [61] applied MRIO-LP for optimizing a fuzzy multi-regional input–output model for biomass supply chain and trade under resources and footprint constraints. Meanwhile, [7] used MRIO-LP for multi-objective optimization to minimize CO_2_ emissions in US’s economy while [62] optimized virtual water trade flows between different regions of a country.

Despite the usefulness of previous research, it is evident that there is limited work on examining how the interactions between these countries can be exploited to help achieve carbon emission reduction targets both collectively and individually in consideration of continued GDP growth rates. This work thus develops a multi-regional input–output (MRIO) based optimization model to determine how a cluster of nations can collectively reduce their carbon emissions in recognition of the individual reduction targets and expected GDP growth rates. The input–output framework provides a structure for representing the interdependence between economic sectors while the MRIO model extends the analysis to account for interactions between the different countries. Transforming the traditional MRIO model into an optimization model helps identify the prescribed economic structure to achieve maximized carbon emission reductions which are useful for crafting policies to mitigate climate change. The rest of this paper is organized as follows. The following section discusses the methodology used starting with a tutorial on the environmentally extended MRIO model. It is then followed by the development of the MRIO based optimization model which can be used for minimizing identified environmental impacts. A motivating example is then presented to illustrate how the model works using a simplified case study. The analysis is then extended to look at countries in the ASEAN region and their interactions with the rest of the world. Then discussion, conclusions, and recommendations for future work are provided.

## Methods

### Environmentally extended multi-regional input–output analysis

Multi-Regional Input–Output models provide a framework for analyzing the economic transactions between regions while keeping the assumptions that are characteristic of IO models (e.g., technical coefficients are constant, changes are immediate, sectors can increase production at any rate, etc.) [41]. The technical coefficient matrix for a 2 region MRIO model is given by Eq. 1 where ${\mathbf{A}}^{\mathbf{r}\mathbf{r}}$ represents the transactions between sectors in region R, ${\mathbf{A}}^{\mathbf{r}\mathbf{s}}$ contains the transactions from region R to region S, ${\mathbf{A}}^{\mathbf{s}\mathbf{r}}$ contains the transactions from region S that are used by region R and ${\mathbf{A}}^{\mathbf{s}\mathbf{s}}$ contains the transactions between sectors in region S. The entries in these matrices, which are of the form ${a}_{ij},$ represents the required inputs from sector I needed to generate a unit of output in sector j. Equation 2 represents the over-all size of sectors in region R (${\mathbf{x}}^{\mathbf{r}}$) and region S (${\mathbf{x}}^{\mathbf{s}}$), the entries of these vectors are of the form ${x}_{j}^{k},$ and is equivalent to the total output of *j* in region *k*. Similar to the basic IO model, the final demand, (**f**) of regions can be obtained using Eq. 3 which can be expanded into Eq. 4. Adding the final demands of each economic sector in a region results in the GDP and represents the amount of goods consumed by households as final goods. Typically, the final demand for products and services are known and the objective is to determine the over-all size of economic sectors so that final demand will be satisfied. In this case, Eq. 5 can be used to solve for matrix **x**.

1 $${{\bf{A}}}=\left[\begin{array}{cc}\bf{{A}^{rr}}& \bf{{A}^{rs}}\\ \bf{{A}^{sr}}& \bf{{A}^{ss}}\end{array}\right]$$

2 $$\bf{x}=\left[\begin{array}{c}\bf{{x}^{r}}\\ \bf{{x}^{s}}\end{array}\right]$$

3 $$\left(\bf{I}-\bf{A}\right)\bf{x}=\bf{f}$$

4 $$\left(\left[\begin{array}{cc}\bf{I}& \bf{0}\\ \bf{0}& \bf{I}\end{array}\right]-\left[\begin{array}{cc}\bf{{A}^{rr}}& \bf{{A}^{rs}}\\ \bf{{A}^{sr}}& \bf{{A}^{ss}}\end{array}\right]\right)\left[\begin{array}{c}\bf{{x}^{r}}\\ \bf{{x}^{s}}\end{array}\right]=\left[\begin{array}{c}\bf{{f}^{r}}\\ \bf{{f}^{s}}\end{array}\right]$$

5 $$\bf{x}={\left(\bf{I}-\bf{A}\right)}^{-1}\bf{f}$$

The MRIO model can be integrated with the environmental IO model to take into consideration the interaction of the economic system with the environment. An optimization model can then be developed to identify potential strategies that can be used to reduce the environmental impact of regions. Equation 6 can be utilized to quantify the over-all environmental impact of the economic system where **B** is the environmental intervention matrix (or direct impact coefficient matrix) and **g** contains the total environmental impact of the system. Equation 6 can be expanded to Eq. 7 to clearly illustrate how each region contributes to the over-all environmental impact.

6 $${{\bf{B}}}{{\bf{x}}}={\bf{g}}$$

7 $$\left[\begin{array}{cc}\bf{{B}^{r}}& \bf{{B}^{s}}\end{array}\right]\left[\begin{array}{c}\bf{{x}^{r}}\\ \bf{{x}^{s}}\end{array}\right]={\bf{g}}$$

Due to the interdependency between economic sectors and between regions, it is expected that meeting the demands of one region will also influence the economic activities of other regions. In a similar way, this will also affect the environmental burden placed on the resources of each region either for meeting their own needs or the need of others. It has been argued that the environmental burden in a region may be reduced by importing resource intensive products rather than utilizing local resources [63]. However, this will obviously shift the environmental burden to another region. A balance must then be made between the environmental impact generated by one region against those generated by another. In this regard, the environmentally extended MRIO model can be formulated into a multi-objective optimization problem where each objective represents the goal of each stakeholder or each region.

### Development of the MRIO optimization model

The MRIO model may be translated into an optimization model to address the given problem statement. The following model assumptions are considered.

- Given *N* number of regions with *M* economic sectors, the interaction between these sectors and between regions is known.
- Given *K* number of relevant environmental emissions, the amount of environmental emission generated corresponding to a level of economic productivity per sector is known.
- Given that each region has a target economic growth
- Given that each region has a target reduction in environmental emission.
- The objective of the model is to determine the final output and final demand of economic sectors which will minimize environmental emissions.

It must be ensured that the MRIO model has degrees of freedom within the system such as the possibility of differentiated sector growth [16]. The objective function can be represented by Eq. 8 which in this case intends to minimize one specific environmental impact, ${g}_{1}$. The optimization model will then be subject to equality constraints as defined by the MRIO model (Eq. 9), environmental impact constraints (Eq. 10) and target growth for each region (Eqs. 11, 12) which must be within reasonable lower (${\mathrm{F}}^{\mathrm{r},\mathrm{L}}, {\mathrm{F}}^{\mathrm{s},\mathrm{L}}$) and upper (${\mathrm{F}}^{\mathrm{r},\mathrm{U}}, {\mathrm{F}}^{\mathrm{s},\mathrm{U}}$) limits. Equation 13 ensures that the capacity of each economic sector is within defined lower (${{\bf{x}}}^{{\bf{L}}}$) and upper limits (${{\bf{x}}}^{{\bf{U}}}$).

8 $$\mathit{min}{g}_{1}$$

9 $$\left({{\bf{I}}}-{{\bf{A}}}\right){{\bf{x}}}={{\bf{f}}}$$

10 $$\bf{Bx}=\bf{g}$$

11 $${\left[{{\varvec{f}}}^{r}\right]}^{T}1\le {F}^{r,U}, {\left[{{\varvec{f}}}^{s}\right]}^{T}1\le {F}^{s,U}$$

12 $${\left[{{\varvec{f}}}^{r}\right]}^{T}1\ge {F}^{r,L}, {\left[{{\varvec{f}}}^{s}\right]}^{T}1\ge {F}^{s,L}$$

13 $${{{\bf{x}}}}^{{{\bf{L}}}}\le {{\bf{x}}}\le \boldsymbol{ }{{{\bf{x}}}}^{{\bf{U}}}$$

### Motivating example

The motivating example used here was taken from the multi-region IO example provided in [41] which was amended with matrix **B** to account for the environmental impacts associated with the economic activities between regions R and S. Note that this is a hypothetical example intended to demonstrate the model developed in this work. Using this framework, it is possible to determine the associated environmental impact from the consumption activities of a region in contrast to environmental impacts resulting from production activities. In this example, region R has 3 sectors while region S has 2. Furthermore, 2 different emissions are considered (e.g. CO_2_ emissions, solid waste). The technical coefficient matrices are given in (14) to (17), these matrices indicate the required input from sector *i* needed to generate a 1 USD output from sector *j*. The final demands for regions R and S are shown in (18) and (19), respectively, these reflect the total amount of products consumed as final goods from sector *i* in region *r*. The associated emission intensity matrices are given in (20) and (21) and represent the amount of emission *k* generated per USD 1 of output from sector *j*.

14 $${{{\bf{A}}}^{rr}}=\left[\begin{array}{ccc}0.1500& 0.2500& 0.0500\\ 0.2000& 0.0500& 0.4000\\ 0.3000& 0.2500& 0.0500\end{array}\right]$$

15 $${{{\bf{A}}}^{ss}}=\left[\begin{array}{cc}0.1667& 0.3125\\ 0.1250& 0.1250\end{array}\right]$$

16 $${{{\bf{A}}}^{rs}}=\left[\begin{array}{cc}0.0208& 0.0938\\ 0.1667& 0.1250\\ 0.0500& 0.0500\end{array}\right]$$

17 $${{{\bf{A}}}^{sr}}=\left[\begin{array}{ccc}0.0750& 0.0500& 0.0600\\ 0.0500& 0.0125& 0.0250\end{array}\right]$$

18 $${{{\bf{f}}}^{r}}=\left[\begin{array}{c}200\\ 1000\\ 50\end{array}\right]$$

19 $${{{\bf{f}}}^{s}}=\left[\begin{array}{c}515\\ 450\end{array}\right]$$

20 $${{{\bf{B}}}^{r}}=\left[\begin{array}{ccc}0.2& 0.3& 0.1\\ 0.1& 0.4& 0.3\end{array}\right]$$

21 $${{{\bf{B}}}}^{s}=\left[\begin{array}{cc}0.3& 0.2\\ 0.2& 0.5\end{array}\right]$$

Taking this example, the matrices can be combined into an MRIO coefficients table as that shown in Table 1 and an emission intensity table as shown in Table 2. The italicized entries represent transactions within the same region. The emission intensity is expressed as the number of units of emission generated per monetary output of an economic sector (e.g. kg CO_2_/million USD). It is possible to determine vector **x** which will satisfy the final demands for regions R and S using Eq. 5. The final demands are satisfied through internal production (e.g. produced within the region) or imports (e.g. produced from another region). Furthermore, the accompanying environmental load can also be obtained using Eq. 6. The results are given below where matrix **x** is shown in (22) while vector **g** is in (23). It is important to note that the resulting **g** accounts for the environmental burden of satisfying demands in both regions R and S. However, it is possible to disaggregate how the resources are allocated across the different sectors and how each sector contributes towards the environmental burden to provide more localized information [64]. This disaggregation is shown in Table 3. However, account for the environmental impact resulting from the demand of the individual regions, the result can be obtained by solving Eq. 9 while using the demand of Regions R and S separately.

**Table 1:** Multi-regional coefficients matrix

	**Region R**			**Region S**		**Demand**
	**1**	**2**	**3**	**1**	**2**	
Region R						
1	*0.1500*	*0.2500*	*0.0500*	0.0208	0.0938	200
2	*0.2000*	*0.0500*	*0.4000*	0.1667	0.1250	1000
3	*0.3000*	*0.2500*	*0.0500*	0.0500	0.0500	50
Region S						
1	0.0750	0.0500	0.0600	*0.1667*	*0.3125*	*515*
2	0.0500	0.0125	0.0250	*0.1250*	*0.1250*	*450*

**Table 2:** Emission intensity of economic sectors (in units of emission/USD)

	Region R		Region S		
	1	2	3	1	2
Emission 1	0.2	0.3	0.1	0.3	0.2
Emission 2	0.1	0.4	0.3	0.2	0.5

**Table 3:** Baseline scenario

	Region R			Region S		Demand	x
	1	2	3	1	2		
Region R							
1	*150*	*500*	*50*	25	75	200	1000
2	*200*	*100*	*400*	200	100	1000	2000
3	*300*	*500*	*50*	60	40	50	1000
Region S							
1	75	100	60	*200*	*250*	*515*	1200
2	50	25	25	*150*	*100*	*450*	800
Total							
Emission 1	200	600	100	360	160		1420
Emission 2	100	800	300	240	400		1840

22 $${{\bf{x}}}=\left[\begin{array}{c}1000\\ 2000\\ \begin{array}{c}1000\\ 1200\\ 800\end{array}\end{array}\right]$$

23 $${{\bf{g}}}=\left[\begin{array}{c}1420\\ 1840\end{array}\right]$$

For example, if the interest is in finding the environmental impact associated with the demands for products of Region R, vector **f** will then be reduced to **f**^r^_A_ = [200 1000 50]^T^, with the values for **f**^s^_A_ = [0 0]^T^ all equal to zero, this is equivalent to the demand column found in Table 4. The resulting allocation of goods and emissions is indicated in Table 4. In a similar manner, it is also possible to obtain the environmental impact associated with the consumption of Region S (where **f**^r^_B_ = [0 0 0]^T^ and **f**^s^_B_ = [515 50]^T^) this is summarized in Table 5. For Table 4, the results obtained for **x**^s^ represents the contribution of production-based activities of Region S to satisfy the demands of Region R while the results obtained for **x**^r^ in Table 5 represents the contribution of production-based activities in Region R to satisfy the demands of Region S. The production-based environmental impact of Region S which are reported in boldface (see Table 4) is thus equal to the generated emissions of the sectors in Region S when it produces products to supply the requirements of Region R and vice-versa. These results clearly show the interdependence between the two regions. In this regard, we can expect that minimizing the environmental impact of one region might result in the increase in impact from the other region due to a potential increase in the amount of traded goods. To demonstrate that reducing the environmental impact of one region does not necessarily reduce the impact of another, we look at the following scenarios, Scenario A considers minimizing Emission 1 of Region R, Scenario B considers minimizing Emission 1 of Region S while Scenario C considers minimizing Emission 1 of the entire system. We make use of the coefficients defined in Tables 1, 2. In all 3 scenarios, we consider that Region R has a target growth rate between 5 and 10% while Region S has a target growth rate of 3–8% for their respective GDPs and that each individual economic sector cannot grow more than 10% from its baseline total capacity. Final demands are also not decreased from the baseline. A summary of the limiting data is shown in Table 6. In addition, Scenario 0 is defined as the state of the economy where the sectors have equal GDP growth of 5% for Region R and 3% for Region S. Equation 8 is modified to Eq. 8a for Scenario A, Eq. 8b for Scenario B and Eq. 8c for Scenario c. Solving for Eqs. 8a, 8b, and 8c subject to the constraints given in Eqs. 9–13, the results of the different scenarios are shown in Tables 7, 8, 9

**Table 4:** Consumption-based transactions and environmental impact for Region R

	Region R			Region S		Demand	x
	1	2	3	1	2		
Region R							
1	*114.67*	*396.03*	*36.57*	5.49	11.70	*200*	764.45
2	*152.89*	*79.21*	*292.54*	43.89	15.60	*1000*	1584.12
3	*229.34*	*396.03*	*36.57*	13.17	6.24	*50*	731.34
Region S							
1	57.33	79.21	43.88	*43.89*	*39.01*	0	263.31
2	38.22	19.80	18.28	*32.91*	*15.60*	0	124.83
Total							
Emission 1	152.89	475.24	73.13	**78.99**	**24.97**		805.22
Emission 2	76.45	633.65	219.40	**52.66**	**62.41**		1044.57

**Table 5:** Consumption-based transactions and environmental impact for region S

	Region R			Region S		Demand	x
	1	2	3	1	2		
Region R							
1	*35.33*	*103.97*	*13.43*	19.51	63.30	0	235.55
2	*47.11*	*20.79*	*107.46*	156.11	84.40	0	415.88
3	*70.66*	*103.97*	*13.43*	46.83	33.76	0	268.66
Region S							
1	17.67	20.79	16.12	*156.11*	*210.99*	515	936.69
2	11.78	5.20	6.72	*117.09*	*84.40*	450	675.17
Total							
Emission 1	47.11	124.76	26.87	281.01	135.03		614.78
Emission 2	23.55	166.35	80.60	187.34	337.59		795.43

**Table 6:** Limiting data for motivating example

Region	Sector	${x}^{L}$	${x}^{U}$	${f}^{L}$	${f}^{U}$
	1	1000	1100	200	NA
R	2	2000	2200	1000	NA
	3	1000	1100	50	NA
	Total	NA	NA	1312.50	1375
S	1	1200	1320	515	NA
	2	800	880	450	NA
	Total	NA	NA	993.95	1042.2

**Table 7:** Scenario A (minimizing Emission 1 for Region R)

	Region R			Region S		Demand	x
	1	2	3	1	2		
R							
1	*153.56*	*513.44*	*54.54*	26.07	76.13	200.00	1023.73
2	*204.75*	*102.69*	*436.30*	208.52	101.50	1000.00	2053.76
3	*307.12*	*513.44*	*54.54*	62.56	40.60	112.50	1090.76
S							
1	76.78	102.69	65.45	*208.52*	*253.76*	543.95	1251.15
2	51.19	25.67	27.27	*156.39*	*101.50*	450.00	812.02
Total							
Emission 1	204.75	616.13	109.08	375.34	162.40		1467.70
Emission 2	102.37	821.51	327.23	250.23	406.01		1907.35

**Table 8:** Scenario B (minimizing emission 1 for region S)

	Region R			Region S		Demand	x
	1	2	3	1	2		
R							
1	*154.05*	*513.78*	*54.63*	25.59	78.95	200.00	1026.98
2	*205.40*	*102.76*	*437.01*	204.70	105.26	1000.00	2055.11
3	*308.10*	*513.78*	*54.63*	61.41	42.10	112.50	1092.51
S							
1	77.02	102.76	65.55	*204.70*	*263.15*	515.00	1228.18
2	51.35	25.69	27.31	*153.52*	*105.26*	478.95	842.08
Total							
Emission 1	205.40	616.53	109.25	368.45	168.42	1468.05	1468.05
Emission 2	102.70	822.05	327.75	245.64	421.04	1919.18	1919.18

**Table 9:** Scenario C (minimizing total emission 1)

	Region R			Region S		Demand	x
	1	2	3	1	2		
R							
1	*153.56*	*513.44*	*54.54*	26.07	76.13	200.00	1023.73
2	*204.75*	*102.69*	*436.30*	208.52	101.50	1000.00	2053.76
3	*307.12*	*513.44*	*54.54*	62.56	40.60	112.50	1090.76
S							
1	76.78	102.69	65.45	*208.52*	*253.76*	543.95	1251.15
2	51.19	25.67	27.27	*156.39*	*101.50*	450.00	812.02
Total							
Emission 1	204.75	616.13	109.08	375.34	162.40	1467.70	1467.70
Emission 2	102.37	821.51	327.23	250.23	406.01	1907.35	1907.35

8a $$\mathrm{min}{g}_{1}^{R}$$

8b $$\mathrm{min}{g}_{1}^{S}$$

8c $$\mathrm{min}{g}_{1}^{R}+{g}_{1}^{S}$$

The summary of the results for Scenario A can be found in Table 7, while those for Scenario B are in Table 8 and that of Scenario C is in Table 9. A summary of the total emissions generated by each region in each of the scenarios presented are shown in Table 10. Table 10 shows that equal GDP growth for all sectors (Scenario 0) results in 1544 units (988 + 556 = 1544) of Emission 1. Emission 1 in Region R achieves the lowest value in Scenario A and C; for Region S, Emission 1 is lowest in Scenario B; and that the entire system can reduce total Emission 1 to 1468 units (Scenario C). This shows how emission tradeoffs can occur when trying to achieve individual emission targets.

**Table 10:** Summary of emission results

	Scenario 0		Scenario A		Scenario B		Scenario C	
	R	S	R	S	R	S	R	S
Emission 1	988.30	555.91	929.95	537.75	931.18	536.87	929.95	537.75
Emission 2	1320.17	682.23	1251.11	656.24	1252.50	666.68	1251.11	656.24

For all three scenarios, Region R achieved a growth rate of 5% while Region S achieved a growth rate of 3%. However, the individual sectors did not grow proportionately. For scenarios A and C, only Sector 3 grew in Region R while Sector 1 grew for Region S. For scenario B, it was also Sector 3 that experienced a growth in Region R while Sector 2 grew for Region S.

Results show that reducing the emissions for one region does not only affect the productivity of that region but also impacts other regions connected to it.

Awareness of the interaction and effect between trading nations may help global communities understand how nations can work together towards defining and meeting their emission targets.

## Results

The method discussed above is used to investigate how the interaction of 12 different regions impact each region’s goal in achieving their nationally determined contributions based on the Paris Agreement. The multi-regional IO table was obtained from the GTAP 10 database and carbon emissions from the use of coal, oil, gas, and other oil products were obtained from the GTAP-E database [66]. The 8 sectors considered in this study is based on the standard GTAP 10 database aggregation of sectors with similar emission levels. Primary focus is given to the ASEAN region. The 12 regions and 8 sectors considered are summarized in Table 11. Note that higher resolution calculations can be implemented if data is available.

**Table 11:** Regions and Sectors considered in the Case study

Regions		Sectors	
R01	Brunei	S1	Agriculture
R02	Cambodia	S2	Coal, oil, gas and oil products
R03	Indonesia	S3	Food
R04	LaoPDR	S4	Transport
R05	Malaysia	S5	Energy intensive industries
R06	Philippines	S6	Non-energy intensive industries
R07	Singapore	S7	Services
R08	Thailand	S8	Electricity
R09	Vietnam		
R10	Rest of South East Asia		
R11	East Asia		
R12	Rest of the World		

### Business-as-usual (BAU) scenario

**Fig. 1:**
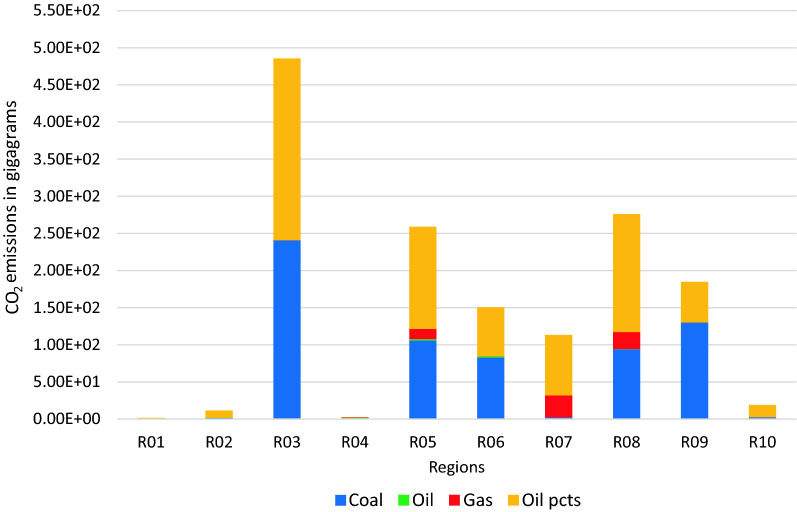
CO_2_ emissions by 2030 for Regions 1 to 10 in gigagrams (BAU scenario)

**Fig. 2:**
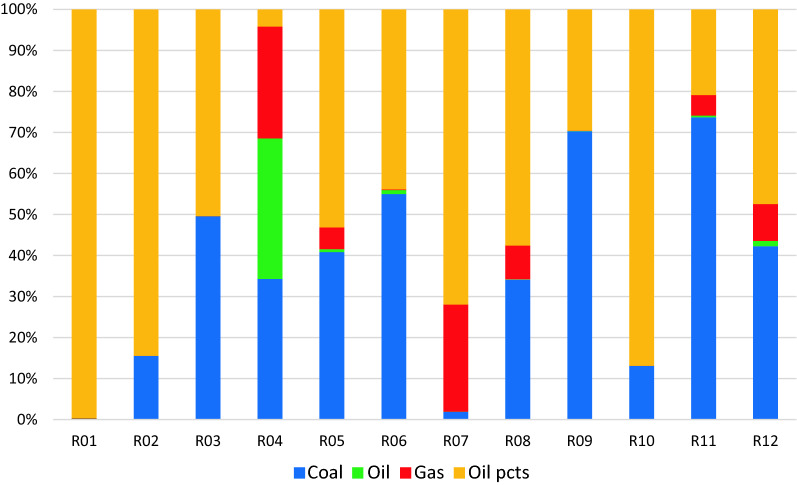
Contribution of energy source to emissions

**Fig. 3:**
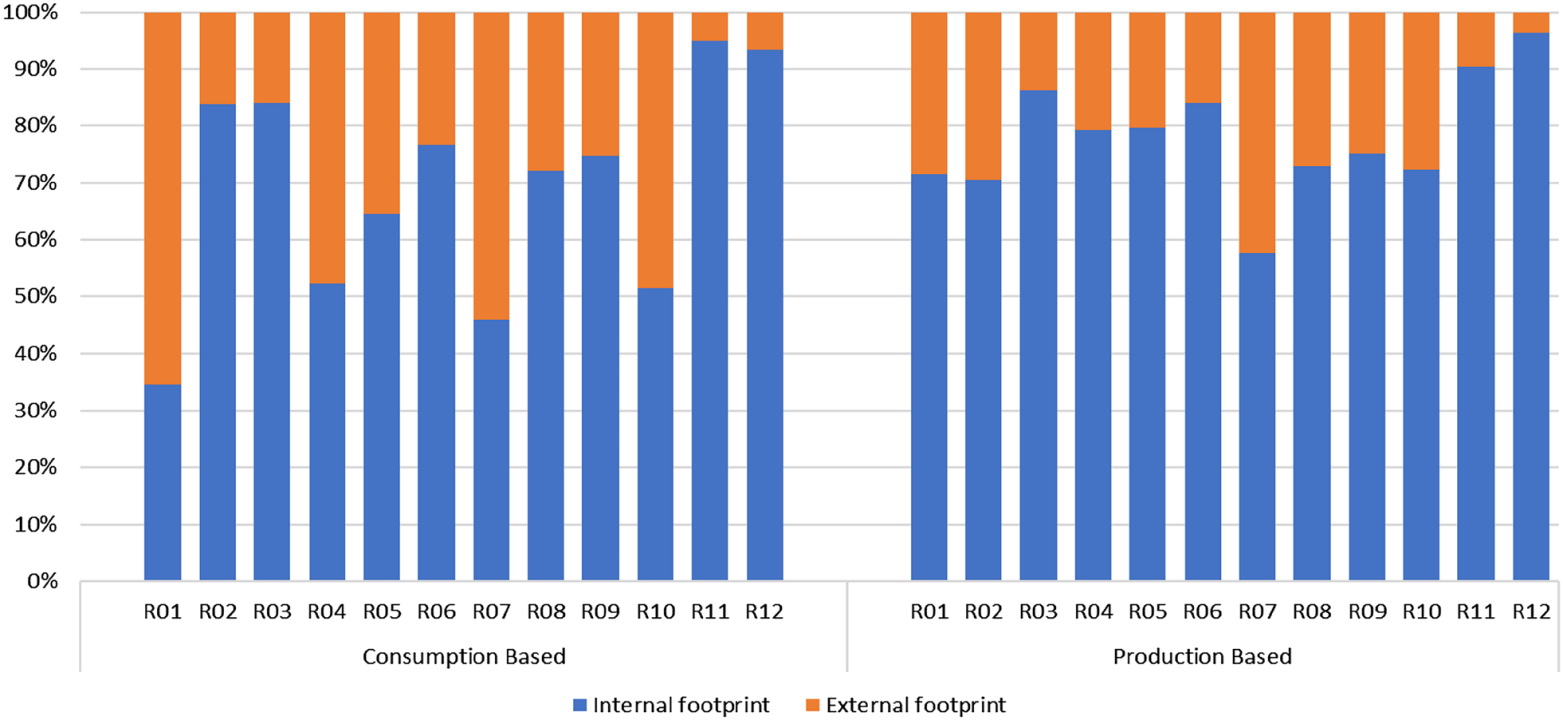
Consumption and Production based contribution of carbon emissions for BAU

Using BAU scenario with the target annual growth rates identified in Table 12, the resulting carbon emissions for the different regions by fuel type is illustrated in Fig. 1 with the relative contributions of the fuel types to over-all carbon emissions shown in Fig. 2. Indonesia has the highest carbon emissions for the countries in Regions 1 to 9, with its emissions almost equally contributed by coal and other oil products. Emissions from coal use had the highest contribution over-all which comprised about 56% of the total. Analysis of the carbon footprint of regions based on their consumption and production patterns are illustrated in Fig. 3. Results show that if we look at the consumption pattern of the regions, Regions 1 and 7 relied heavily on imports such that carbon emissions generated to support their demand were primarily (i.e. more than 50%) generated in another region. The carbon emissions of the remaining regions were generated primarily (i.e. more than 50% of emissions) from their own economic activities. Alternatively, looking at the production-based emissions, Region 7 had the highest carbon emission proportion associated with activities meant to support the demand of other regions.

**Table 12:** Target growth rates and NDCs for 2030

	Average annual growth in GDP from 202–2030 (in %)	Carbon emission reductions by 2030 from BAU (in %)
R01		
Brunei	2.60	
R02		
Cambodia	7.16	
R03		
Indonesia	5.36	29.00
R04		
LaoPDR	5.62	
R05		
Malaysia	5.96	45.00
R06		
Philippines	6.66	70.00
R07		
Singapore	3.04	
R08		
Thailand	4.14	20.80
R09		
Vietnam	6.96	9.00
R010		
Rest of South East Asia (RoSEA)	4.06	
R011		
East Asia	5.12	74.00
R012		
Rest of the World (ROW)	4.06	45.00

### Scenario 1–differentiated growth

We now considered the scenario wherein differentiated sectoral growth is allowed in each region if the over-all national growth rate was still greater than the target average annual growth as indicated in Table 12. This contrasts with the assumption that all economic sectors will grow at the same rate. Such a scenario can be realized by infusing more funds into priority sectors. Each sector in the regions could contract or grow by ± 10% from the target rate. Solving Eqs. 8–13, the resulting growth rate for the individual sectors per region considered is shown in Table 13. Entries which have been marked with red downward arrows indicate that the annual growth rate of the associated sector in each region is below the average target growth rate of the region. A green upward arrow indicates that the sector had a higher growth rate compared to the average target growth rate of the region. Sector 7 (Services Sector) in almost all regions grew more than the target growth rate except in the case of Cambodia and the Philippines. For Cambodia and the Philippines, the service sector has the largest share of their gross domestic product at 36.60% and 61.42% respectively [66]. Sectors 2, 4, 5, 6 and 9 mostly had growth rates below the regional target growth rates indicating that these sectors should not be prioritized if the intention is to meet carbon reduction targets, since these sectors had higher carbon intensity.

**Table 13:**
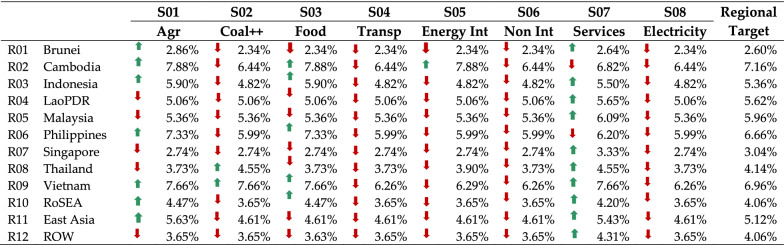
Annual growth rates of different sectors across the different regions

**Fig. 4:**
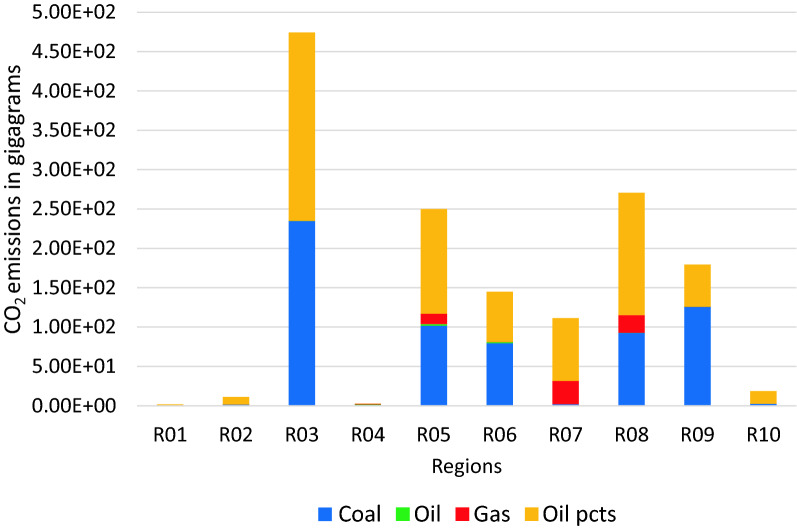
Contribution of fuel source to emissions (Scenario 1)

**Fig. 5:**
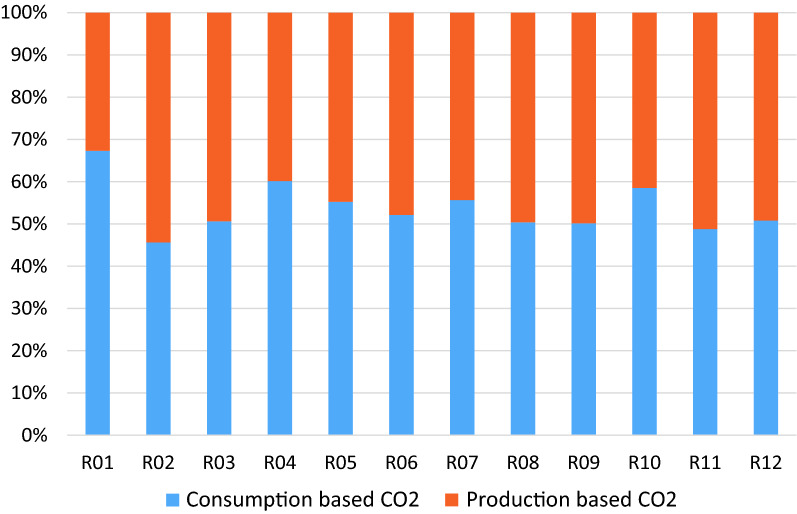
Consumption and Production based contribution of carbon emissions for Scenario 1

**Fig. 6:**
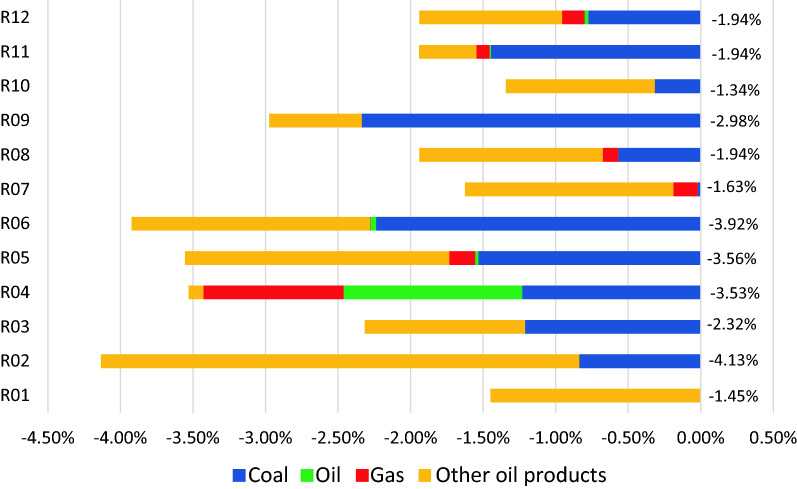
Change in CO_2_ emissions from BAU to differentiated growth

This differentiated growth achieved almost a 2% over-all reduction in carbon emissions in comparison to the BAU scenario with the contribution of each region illustrated in Fig. 4. Figure 5 on the other hand shows how the emission for each region is accounted for using a consumption based and production-based perspective. Figure 6 illustrates the reduction in CO_2_ achieved in each region and where the reductions were realized with regards to the fuel used. All regions were able to reduce their emission levels while meeting the desired GDP growth rates. If only individual countries were analyzed, Region 2 achieved the highest reductions in carbon emissions at 4.13%, the reductions were from the decrease in emissions of other oil products and of coal which arose from reduced growth of sectors reliant on these types of fuel. Region 1 had the lowest reductions in emissions. It can be noted that Region 1 (Brunei) does not have coal-fired power plants, however, Brunei also has to exert much effort in harnessing renewable energy in the electricity generation aspect as the solar energy remains to be their sole renewable energy resource [67]. None of the countries achieved the target carbon emission reductions as indicated in Table 12. Customized regional strategies can be developed based on the results shown in Table 13 to ensure that the reductions obtained from the model results are indeed achieved. Focus can be given on sectors which provide the highest economic growth potential since growth in these sectors can be accommodated despite identified reduction targets. In the Philippines for example, policies which support the growth of the agricultural and food industry which were recommended to have higher growth rates of 7.33%, should be strengthened.

### Scenario 2 reduced carbon intensity in the electricity sector

**Fig. 7:**
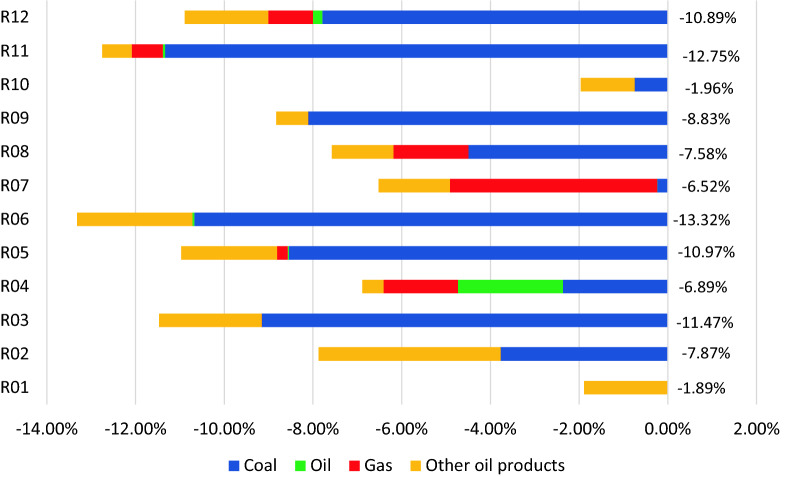
Change in CO_2_ emissions from BAU to reduced carbon intensity of the electricity sector

The next scenario couples differentiated growth rates with a 20% reduction in carbon intensity of the electricity sector in all regions. This is a conservative estimate following the Doha Amendment to the Kyoto Protocol which aimed to reduce the greenhouse gas emissions of the European Union by 20% [68]. This scenario is possible if a country decides to increase renewable energy penetration in its national grid. The ASEAN economies have developed their programs to reduce their dependence on non-renewable resources and shift towards renewable energy into their primary energy mix. For example, the Indonesia Energy Law of 2007 was enacted to reduce the import dependence on refined oil. In the Philippines, the Renewable Energy Act of 2008 seeks to promote the development of the renewable energy sector of the country. In Malaysia, the Renewable Energy Act of 2011 is also in place. While these legislations have been in place for quite some time, it is only recently that the technological innovations have made it more efficient to invest in the sector. Results suggest that it is possible to reduce the carbon footprint further to 11.6%. The reductions for each region in comparison to the BAU scenario are illustrated in Fig. 9. In this case, Region 6 (Philippines) achieved the highest reduction in carbon footprint while Region 1 (Brunei) achieved the least. Figure 7 also shows that various countries efforts in veering away from coal, Region 07 (Singapore) will have a significant reduction in its gas consumption as a result, given that 95% of their electricity is generated using natural gas [69].

It is worth noting that this 20% reduction serves as an illustrative benchmark at the strategic planning level for policymakers to analyze the requirements in achieving it. However, this target must be analyzed further in future work to understand its practicality based on detailed operational rollout.

## Discussion

The scenarios presented for the MRIO model which consisted of 9 individual countries, rest of Southeast Asia, East Asia, and the rest of the world demonstrates how individual carbon emissions reductions of nations are linked with each other. Differentiated sector growth rates and reductions in the carbon intensity of the electricity sector are some strategies which can be considered to achieve carbon emission reductions. In both cases, carbon emission reductions were achieved both collectively and for individual regions (e.g., countries) without greatly varying the current level of interdependence between regions. This means that the production structure of each economic sector remains the same. It can be seen however, that certain countries achieved higher reductions therefore compensating for lower reductions achieved in other nations. These results were influenced by the existing economic structure of individual nations. In this regard, more customized policies can be developed in each country to realize higher carbon emission reductions while maintaining target GDP growth rates. For example, Indonesia had the highest expected CO_2_ emission by 2030 for the BAU case. Implementing differentiated growth results in a 2.32% reduction in emissions compared to BAU, however, implementing a reduction in the carbon intensity of the electricity sector can further bring down Indonesia’s carbon emissions by 11.47% from BAU. It is possible to continue the growth in the economic sectors with higher growth rates in the sectors of agriculture, food, and services by increasing expenditures in these sectors. Crafting of international policies should take trade structures in consideration when crafting regional emission reduction targets. For example, based on the results of the case study, the agricultural sector should be encouraged only for certain countries (e.g. Brunei, Cambodia, Indonesia, Philippines, Vietnam) and that other sectors should be encouraged in other countries. Such changes can initiate other transformations such as developing competencies and establishing new trade agreements. In addition, the developments from the Conference of Parties held last November 2021 (COP26) in Glasgow highlights the importance of climate financing that can extend the benefits of reducing the carbon emissions of a region to reduced carbon emissions across the supply chain.

It is important to note that the MRIO model used in this work is an extension of the basic IO framework and thus assumes that the economy is in equilibrium and that the technology remains constant (i.e. constant technical coefficients). Though it is a simplified representation of the economy, its essential feature is in capturing the interdependencies between economic sectors which makes it an effective tool for estimating direct and indirect effects of positive and negative shocks on an economy. The results obtained from the scenario analysis reflect the interdependencies between the components considered and thus reveal how the entire system would react if changes were instituted in one or multiple regions (e.g. increase in renewable energy penetration within a nation’s energy mix).

## Conclusions

A multi-regional IO-based optimization model for reducing global carbon emissions has been developed in this work. This model can be utilized for assessing the potential carbon emission reductions that can be achieved given the adoption of certain strategies. This model provides a more holistic view of how the generation of carbon emissions are influenced by the interdependence of nations. The results obtained from the different scenarios considered minimized the collective carbon emissions for the 12 regions used in the analysis. However, the reductions achieved by the regions varied between each other which reflects the state of technology and the level of economic development in the different regions. This approach can thus be used to help nations identify more appropriate and achievable carbon reduction targets as well as develop more customized policies to target priority sectors in a country. However, one of the limitations of the model is that it made use of fixed coefficients to represent the exchange and interdependence of different regions. As a result, the structure for multi-regional trade is not flexible. Future work can thus investigate modelling flexible multi-regional trade where regions have the option to select where to import or export goods from or to consider substitutability of goods and products. More complex models which make use of non-linear relationships between economic parameters can be developed using computable general equilibrium (CGE). Other carbon reduction strategies can also be considered in the scenarios such as reductions in the carbon emission intensity within the transport sector. In addition, governments can explore shifting to alternative energy sources that can address the energy demand of their respective countries and at the same time achieve their emission reduction targets. Finally, hybrid models which integrate the MRIO–LP model with DEA can be explored in the future.

## Data Availability

The dataset used in this study is proprietary data accessed from the Global Trade Analysis Project. The models developed in LINGO may be accessed at https://github.com/kbaviso/ASEAN_MRIO/tree/main.

## Abbreviations

ASEAN: Association of Southeast Asian Nations
BAU: Business as usual
CGE: Computable general equilibrium
CO_2_: Carbon dioxide
COP26: Conference of parties 2026
DEA: Data envelopment analysis
DMUs: Decision-making units
GDP: Gross domestic product
GHG: Greenhouse gas
GTAP: Global trade analysis project
IO: Input–output
IPCC: Intergovernmental panel on climate change
LMDI: Log mean divisia index
LP: Linear programing
LULUCF: Land use, land-use change, and forestry
MRIO: Multi-regional input–output
NDC: Nationally determined contributions
RE: Renewable energy
